# Two Cases of Successful Cæsarean Section

**Published:** 1854-10

**Authors:** Wm. H. Merinar

**Affiliations:** Oktibbeha County, Mississippi


*Two Cases of successful Cæsarean Section.* By WM. H. MERINAR, M. D., of Oktibbeha County, Mississippi.

Phebe, æt., 24 years, servant girl of Mr. Wm. H. Glenn, of this county, was taken in labor on the 14th day of July, 1852. A female was called to her assistance, who attended her until the evening of the 17th inst. Finding that the labor did not advance, though the pains were most violent, she requested that I might be sent for. Upon my arrival, at 9 o'clock, P. M., I was informed that the labor had been active from the beginning, and that the waters had escaped the day before. I found on examination the os uteri sufficiently dilated to introduce a finger and with difficulty touch the fœtal head, which still rested above the pubis. By careful examination, I discovered that there was great deformity of the pelvis—its antero-posterior diameter did not exceed two inches.

I informed Mr. Glenn that delivery per vias naturales could not be hoped for, and requested a consultation with Dr. C. P. Montgomery, who was immediately sent for.

At 8 o'clock, on the morning of the 18th, Dr. M. arrived, and, after repeated examinations, fully concurred with me in opinion. There had been no contractions since three o'clock in the morning; the patient lay exhausted and stupid, skin hot and dry, great tenderness over the abdomen, thirst urgent, pulse 124.

We at once decided that Cæsarean section was our only resort, as the position of the child rendered any other operation impracticable, and I agreed to undertake the operation. The condition of the patient being described to her, she, after considerable reflection, and conversation with her husband and father, consented to submit to whatever we thought best for her.

The necessary arrangements having been made, I had a mattress and sheets fixed upon a firm bedstead, and, the head and shoulders being well supported by pillows, I pinched up a fold of the skin and muscles, about three quarters of an inch from, and on a line with the lower edge of the umbilicus, and passed a curved bistoury, with the back downward, through the parts to the peritoneal sac at once. The intestines being held back by Dr. Montgomery, I carried the incision—about five and a half inches in length with a slight curve—to the linea alba, an inch and a half above the symphysis pubis. The womb being exposed, an incision was made through its walls in the same way.

There was a left lateral obliquity of the uterus, and the child corresponded with the second vertex presentation of Baudeloque. The membranes having been ruptured two days before, I introduced my left hand, and, drawing the head to the orifice, delivered a large, but dead, male child. The placenta and membranes were then carefully removed. The womb contracted imperfectly, though sufficiently to prevent serious hæmorrhage.

No vessel requiring a ligature having been divided, I drew the edges of the wound carefully together, and with three silk ligatures, in the form of interrupted sutures, secured them, and gave additional support with adhesive straps, a compress and bandage. I then applied the cold water dressing, and ordered tinct. opii gtt. xlv.

July 19, 7 o'clock A. M.—I visited her in company with Dr. Montgomery. She spent the most of the night quietly, but with symptoms still alarming; bowels moved twice since I left her. Ordered pulv. opii gr. ii., to be repeated if necessary. Water dressing continued.

4 o'clock, P. M.—Lochia established. Other symptoms the same.

July 20, 6 o'clock, A. M.—Rested quietly through the night; skin still hot and dry; thirst urgent; considerable tympanites; pulse 124. Ordered, aquæ calcis ℥ii. every two hours. Flaxseed tea advised in place of water.

5 o'clock, P. M.—No change since morning.

July 21, 8 o'clock, A. M.—Rested but little through the night; mind not so clear this morning as usual; tongue contracted and red; skin hot and dry; thirst distressing; pulse 130. Ordered bitart. potass., a teaspoonful; to be repeated if necessary.—Water dressing continued, with a cold towel on the forehead.

4 o'clock, P. M.—Bowels moved twice; mind clear but anxious; less heat of skin; pulse 124. Cold applications to the head and bowels continued.

July 22, 7 o'clock, A. M. Rested better last night than usual, but complains of severe pain, since 3 o'clock in the morning, in the right hypochondrium; other symptoms the same. Ordered ℞. pulv. opii, gr. ii, ipecac. pulv., hyd. chlor. mit., *aa.* gr. ss. every two hours, with fomentations over the seat of pain.

4 o'clock, P. M.—Pain relieved after the second pill; thirst still urgent; has taken no nourishment since the morning of the 17th.

July 23d, 8 o'clock, A. M.—Rested well during the night; pulse still 124; other symptoms more favorable.

July 24, 9 o'clock, A. M.—Symptoms more favorable; bowels moved once; mind clear and tranquil; thirst subsiding; pulse 118; wound examined; discharge healthy.

July 25, 8 o'clock, A. M.—Slept comfortably until 3 o'clock this morning, when she was disturbed by pains in the bowels, which were relieved by tinct. opii gtt. xlv.; skin soft; thirst subsiding; mind relieved; pulse 112.

July 26, 8 o'clock A. M.—Symptoms improving; has taken a few spoonsful of gruel this morning; pulse 108.

July 27, 9 o'clock, A. M.—Rested well through the night; bowels moved twice; appetite improving; other symptoms favorable; granulations healthy; sutures removed.

July 28, half-past 10 o'clock A. M.—Rested well all night; pulse 104.

July 29, 9 o'clock, A. M.—No change since my last visit; one evacuation from the bowels.

July 30, 8 o'clock, A. M.—Rested well; spent the night comfortably; bowels moved once; skin soft; tongue cleaning off; thirst subsiding; appetite improving; spirits raised with the hope of recovery; pulse 94.

August 2, 9 o'clock, A. M.—Symptoms improving; pulse 88.

August 5, 9 o'clock, A. M.—Rested badly through the night, from imprudence in diet and neglect of bowels; pulse 106. Ordered ol. ricini, a tablespoonful.

4 o'clock, P. M.—Oil moved twice; symptoms all better; pulse 90.

August 8, 8 o'clock, A. M.—Still improving; pulse 78; wound healthy; new dressing applied; but little soreness complained of.

August 13, 8 o'clock, A. M.—No material change since my last visit; pulse 74.

August 20, 9 o'clock, A. M.—Symptoms all favorable; granulations healthy; pulse 70.

August 26, 4 o'clock, P. M.—Still improving; wound healed, except an inch at the lower edge.

September 14, 9 o'clock, A. M.—Wound entirely closed; sits up an hour or two at a time; says she feels no pain or inconvenience from the operation; bowels regular; appetite good.

September 30, 8 o'clock, A. M.—Enjoying perfect health, and attends to her business as usual.

On the 22d of May, 1854, I was called to visit the unfortunate individual whose case I have just detailed, in labor again. Upon my arrival, at 9 o'clock, P. M., I found the pains frequent, and the most violent that I ever witnessed.

I at once requested a consultation with Dr. Montgomery, who was sent for but could not attend, his own family being indisposed. Other physicians were sent for but could not be had.

The pains continued so violent—notwithstanding opiates were freely used—as to threaten danger, and at midnight I determined, with the assistance of Mr. Glenn, again to perform the Cæsarean section. The patient urged me not to wait longer for consultation.

I had a mattress and sheets fixed upon a firm bedstead, and the patient placed upon her back, with her head and shoulders supported by pillows, as in the first case. I then pinched up carefully a fold of the skin and muscles, about midway between the old cicatrix and umbilicus, and, passing a scalpel with the back downwards, cut a small orifice through to the peritoneal sac at once. I then directed Mr. Glenn to press the bowels back by placing one hand on each side of the incision. Carefully introducing a director, and placing the point of the scalpel in the groove, I carried my incision parallel with the original one, until it terminated in the linea alba. The womb being exposed, I made a small incision very carefully through its walls, and passing the director to serve as a guide and protect the membranes from the point of the instrument, I made the orifice to correspond with the one in the abdomen. I then directed Mr. Glenn to press the walls of the abdomen down against the uterus, with sufficient force to cause the membranes to bag externally, and divided them; avoiding, as far as possible, the escape of the liquor amnii into the peritoneal sac.

There was a right lateral obliquity of the uterus, and the child was found to correspond with the first vertex presentation of Baudeloque. I introduced my right hand and passing it carefully around the head, delivered a well grown male child, Cæsar Augustus, who greeted his anxious and distressed mother with the usual salutation. The placenta and membranes were then removed. There was but slight hemorrhage, the womb contracting violently.

I drew the edges of the incision in the abdomen carefully together, and secured them with four silk sutures—interrupted—and gave the necessary support by adhesive straps, a compress and bandage. The cold water dressing was applied, and pulv.

opii. gr. ii. ordered. She became easy in half an hour, and spent the night comfortably.

May 21st, 2 o'clock, A. M.—Dr. S. J. Williams present; resting quietly, skin soft and cool, pulse 96.

2 o'clock, P. M.—No change since morning.

May 22, 8 o'clock, A. M.—Spent the night badly, mind anxious, skin warm and dry, considerable thirst, discharged urine twice, but no action from the bowels, pulse 108. Ordered bitart. potassæ, a teaspoonful every two hours until it acts.

4 o'clock, P. M.—Symptoms the same; no evacuation from the bowels. Ordered ol. ricin., a tablespoonful.

May 23d, 8 o'clock, A. M.—Drs. John McMillan and John M. Rogers present. Spent the night comfortably; lochia established, skin soft, thirst subsiding, mind relieved, appetite good, bowels moved twice, pulse 104, child doing well.

May 24th, 8 o'clock, A. M.—Drs. J. W. Caldwell and J. McMillan present. Slept well except when disturbed by the child, bowels moved once, skin soft, takes nourishment, still cheerful, pulse 100.

May 25th, half past 10 o'clock, A. M.—Rev. J. M. Jones present. Rested well since my last visit; one evacuation from the bowels, pulse still 100.

May 26th, 8 o'clock, A. M.—Rested well through the night, but had this morning a difficulty with her nurse, which produced considerable fever and pain in the bowels, pulse 120. Ordered tinct. opii camph. ʒii., spts. nitre dulc. ʒi. every two hours; cold water continued.

May 27th, 9 o'clock, A. M.—Drs. Rogers and McMillan present. Passed a restless night, skin hot and dry, thirst distressing, mind anxious, violent pain in the region of the liver, no action from the bowels, pulse 126. Ordered ℞. pulv. opii gr. ii., ipecac. pulv., hyd. chlor. mit. *aa.* gr. ss., every two hours with a blister over the pain.

4 o'clock, P. M.—Resting more quietly, but no change in symptoms. Ordered ol. ricini. a tablespoonful.

May 28th, 9 o'clock, A. M.—Oil operated twice, symptoms improved, blister drew well, pain relieved, pulse 114. Wound examined, suppurating sufficiently to produce some smell; dressing renewed, water continued.

May 30th, 9 o'clock, A. M.—Passed the night pleasantly, bowels moved once, appetite good, secretion of milk more abundant, pulse 116.

June 1st, 9 o'clock, A. M.—Rested well through the night, two evacuations from the bowels, no pain and but little soreness, appetite good, adhesive straps renewed, wound still healthy.

June 3d, 4 o'clock, P. M.—Symptoms still favorable, pulse 96, sutures removed.

June 6th, 8 o'clock, A. M.—Still improving, bowels regular, appetite good, pulse 78, Cæsar Augustus still doing well.

June 9th, 11 o'clock, A. M.—Symptoms all favorable, pulse 72.

June 13th, 8 o'clock, A. M.—Doing well, adhesive straps renewed, granulations healthy, water dressing continued.

June 18th, 8 o'clock, P. M.—Mother and child doing well; wound almost healed.

June 28th, 8 o'clock, A. M.—Wound entirely healed, general health good, wants to be up.

July 16th.—Enjoying perfect health, and says she feels no pain nor inconvenience from the operation.

Aug. 28th.—It is now more than three months since the operation was performed. Mother and child enjoy fine health, and she attends to her business as usual.

